# Supplementary material for: A Humanized Yeast Phenomic Model of Deoxycytidine Kinase to Predict Genetic Buffering of Nucleoside Analog Cytotoxicity
Source: Genes (Basel). 2019 Sep 30;10(10):770. doi: 10.3390/genes10100770 (PMC6826991; doi:10.3390/genes10100770)
Supplement: Supplementary file 1 [file genes-10-00770-s001.zip › Additional_Files/Additional_File7_GOTermHeatmaps.zip!/Additional_File7_GOTermHeatmaps/histone_methyltransferase_activity_(H3-K4_specific).pdf]

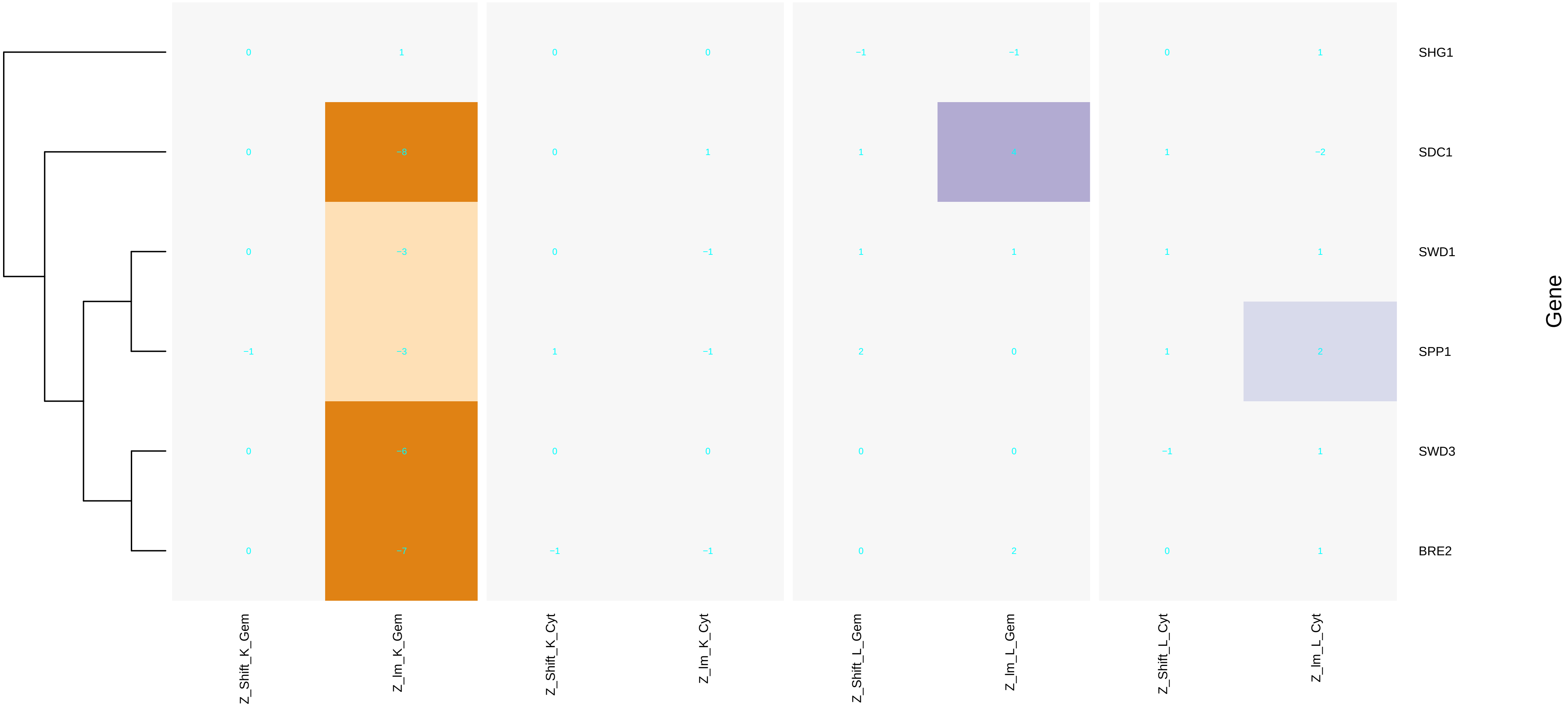

0 1 0 0 −1 −1 0 1
0 −8 0 1 1 4 1 −2
0 −3 0 −1 1 1 1 1
−1 −3 1 −1 2 0 1 2
0 −6 0 0 0 0 −1 1
0 −7 −1 −1 0 2 0 1
SHG1
SDC1
SWD1
SPP1
SWD3
BRE2
Gene
Z_Shift_K_Gem
Z_Im_K_Gem
Z_Shift_K_Cyt
Z_Im_K_Cyt
Z_Shift_L_Gem
Z_Im_L_Gem
Z_Shift_L_Cyt
Z_Im_L_Cyt
